# A comparative study of the prevalence of and factors associated with insecticide-treated nets usage among children under 5 years of age in households that already own nets in Malawi

**DOI:** 10.1186/s12936-019-2667-4

**Published:** 2019-02-20

**Authors:** Owen Nkoka, Martha Sinya Chipeta, Ying-Chih Chuang, Deleon Fergus, Kun-Yang Chuang

**Affiliations:** School of Public Health, Taipei Medical University, 250 Wu-Hsing Street, Taipei, 11031 Taiwan

**Keywords:** Malaria, Insecticide-treated nets, Malawi, Prevention

## Abstract

**Background:**

Despite malaria control programmes having successfully increased the number of households owning insecticide-treated nets (ITNs) in Malawi, the population of people with ITN access but still not using them fluctuated from 13% in 2010, 5% in 2012 and then 12% in 2015. This study aimed to compare the rate and factors associated with ITN usage among children under 5 years of age, living in household with at least one ITN, in Malawi between 2010 and 2015.

**Methods:**

The 2010 and 2015–2016 Malawi Demographic and Health Surveys (MDHSs) were utilized. Only children from households that owned at least one ITN were selected. Multivariate logistic regression analyses were performed to examine associations of child, maternal and household factors with ITN usage.

**Results:**

In total, 12,378 and 10,196 children under 5 years of age were examined from 2010 and 2015–2016, respectively. ITN usage increased from 57.8% (95% Confidence interval (CI): 56.1%–59.4%) in 2010 to 69.0% (95% CI 67.4%–70.5%) in 2015. The multivariate analysis revealed that, among others, being aged ≥ 24 months, having mothers with no formal education or with primary education, residing in a female-headed household, and residing in households that had poor household ITN supply were significantly associated with reduced odds of ITN usage.

**Conclusions:**

ITNs are a key vector control intervention in malaria prevention. This study revealed increased ITN usage among children under 5 years old in the 5-year period, suggesting that considerable improvements have been made. However, continued efforts to increase awareness of the importance of using ITNs in malaria prevention in Malawi are necessary. Findings from this research provide some policy implications, especially for improving household ITN supply, to improve ITN utilization in Malawi.

## Background

Malaria is one of the leading causes of death in sub-Saharan Africa (SSA) [1]. The disease is caused by the *Plasmodium* parasite and is transmitted by the female *Anopheles* mosquito [2]. One of the most important efforts in malaria eradication is the vector control approach which aims at interrupting malaria transmission [1]. Indoor residual spraying with an insecticide to kill mosquitoes, and the use of insecticide-treated nets (ITNs) to prevent mosquito bites are recommended for most of SSA countries. In particular, ITNs are a cost-effective intervention [3–6], and evidence suggests that they are responsible for a 50% reduction in the malaria incidence in malaria-endemic countries [6]. Despite increased efforts to improve ITN accessibility, approximately 437,000 children still die from malaria in Africa every year [7]. The World Health Organization states that the African region is the hardest hit by malaria. For instance, 92 and 90% of all malaria cases and deaths in 2016 worldwide occurred in this region [1]. Additionally, most of these deaths occurred in children under the age of 5 years [1].

Malaria is endemic in Malawi, and there is a huge burden of the disease among children under 5 years old [8]. Malawi experiences perennial malaria transmission with peaks observed in the rainy season between November and April [9]. In 2009, 34% of all outpatient visits, and 40% of all hospitalizations for children under 5 years of age were due to malaria [10, 11]. In an attempt to control malaria transmission, in 2001, Malawi was the first country in SSA region to initiate a nationwide ITN social marketing programme [11]. The programme successfully increased ITN ownership and access from 27% and 19% for ownership and access in 2004 to 57% and 38% for ownership and access in 2010, respectively [12]. According to the Roll Back Malaria Monitoring and Evaluation Reference Group, ITN access refers to the proportion of the household population that could have slept under an ITN, assuming each ITN is used by two people and ITN ownership is defined as the proportion of households with at least one ITN [13]. Yet despite the increase in ITN ownership and access, the malaria incidence did not significantly decrease among children [10]. The slow decline in malaria incidence might be attributed to low usage of ITNs among those owning ITNs [12]. Further, a nationwide ITN distribution campaign was conducted in 2012 which increased household ownership of at least one ITN from 58% in 2010 to 70% in 2014 [14]. Subsequent to the 2012 nationwide campaign, there are limited nationally representative studies that have examined ITN usage among children under 5 years in Malawi.

This study examined factors associated with ITN usage among children under 5 years who live in households that already own an ITN in Malawi between 2010 and 2015–2016. Specifically, using nationally representative samples, the study compared ITN usage, and associated factors before and after the nationwide ITN distribution campaign conducted in 2012.

## Methods

This was a cross-sectional study that utilized data from the 2010 and 2015–2016 Malawi Demographic and Health Surveys (MDHSs). Datasets used for this study, and their documentation can be downloaded, upon request, from https://dhsprogram.com/data/available-datasets.cfm. Briefly, both the 2010 and 2015–2016 MDHSs used the 2008 Malawi Population and Housing Census (MPHC) as the sampling frame [12, 15]. The surveys used a stratified, two-stage, cluster sampling method to select nationally representative samples. The first stage involved selection of enumeration areas (EAs), as defined by the MPHC. The second stage involved selection of households in each EA using probability systematic selection. In 2010, totals of 20 and 35 households from rural and urban clusters were selected, respectively, while 30 and 33 households from urban and rural clusters, respectively, were selected in 2015.

Data were collected through face-to-face interviews from June to November 2010 for the 2010 survey and from October 2015 to February 2016 for the 2015–2016 survey [12, 15]. The survey questionnaires collected information on the mother’s demographics, children’s illnesses, ITN use, etc. from women aged 15–49 years [13]. The current analysis was restricted to children under 5 years old from households that owned at least one ITN. In this study, data from both the household and mother questionnaires were used.

### Variables

#### Outcome variable

The outcome variable was ITN usage defined as whether a child under 5 years of age, living in a household that owned at least one ITN, had slept in an ITN the previous night before the survey. This was assessed as a categorical variable (yes/no).

#### Independent variables

Three sets of independent variables were considered in the analysis, namely child factors, maternal characteristics and household factors. Child factors included age in months (≤ 12, 13–23, ≥ 24), and gender of the child (male and female). Maternal characteristics included age in years (15–24, 25–34, ≥ 35), educational level (no formal education, primary, secondary/post-secondary), employed (yes, no), ethnicity (Chewa, Tumbuka, Lomwe, Yao, Ngoni, others), marital status (married, unmarried), religion (Catholic, Protestant, Muslim, others), women’s health behaviours (undesirable vs desirable). Women’s health behaviour was measured using two constructs; number of antenatal care visits (< 4 vs ≥ 4) [16] and baby postnatal check within 2 months (no vs yes). The composite score from the two dimensions ranged from 0 to 2, with 0 (none of the two services) and 1 (one of the two services) representing undesirable health behaviour, and 2 (those who confirmed to have used the two services) representing desirable health behaviours. Finally, household factors included the gender of the head of the household (male, female), roofing materials (improved: cement and roofing shingles; unimproved: no roof, thatch/palm leaf, sod, rudimentary, rustic mat, palm/bamboo, wood planks, cardboard, wood, others) [17], region (northern, central, southern), and residence (urban, rural). A principal component analysis (PCA) model was used to measure household wealth from a set of materials owned by a household such as bicycles, televisions, etc. Scores were then grouped into quintiles with the upper 20% categorized as richest, the next 20% as richer, middle 20% as middle, penultimate 20% as poorer, and lower 20% as poorest. Household ITN supply was defined as ITN: member ratio within the household [13]. This variable was categorized as very poor (where more than 3 household members had access to 1 ITN), poor (where exactly 3 household members had access to 1 ITN) and good (where exactly 2 household members had access to 1 ITN or where the household had more ITNs than the number of household members).

### Statistical analysis

Pooled data from the two survey waves were first analysed to compare differences in the distributions of socio-economic factors between the two surveys using a Chi squared (χ^2^) test. Thereafter, the survey waves were separately analysed. Further, χ^2^ tests were used to examine the relationship between socio-economic factors and ITN utilization. Associations of child, maternal, and household characteristics with ITN non-use were examined using a multivariate logistic regression. Results from the multivariate logistic regression were reported as the adjusted odds ratio (aOR) and 95% confidence interval (CI). The level of statistical significance was set at 5%. All analyses took into consideration the complex sample design of the MDHS by applying the “svy” command. The svy command accounts for sampling weights and clustering effects in the dataset. All analyses were performed using Stata vers. 15.0 (Stata Corp., College Station, TX, USA).

### Ethical considerations

The procedures and questionnaires for the standard demographic health survey (DHS) surveys were reviewed and approved by the International Classification of Functioning Disability and Health Institutional Review Board, and ethical clearance was obtained from the Malawi Health Sciences Research. Informed consent was obtained at the start of each interview. Permission to use of the data was sought from the DHS programme.

## Results

### Distribution of participants by survey year

Totals of 12,378 and 10,196 children under 5 years old were analysed in 2010 and 2015–2016, respectively. Differences between the 2010 and 2015–2016 surveys were significant in terms of the ages of the children, maternal ages, educational levels, employed, marital statuses, health behaviour, gender of the head of the household, and roofing materials (all *p *< 0.05) (Table 1). There were no significant differences between the remaining factors between the two survey years.

**Table 1:** Description of sample characteristics

Variable	2010 *n* = 12,378		2015–2016 *n* = 10,196		Comparison *p* value
	*n*	%	*n*	%	
Child factors					
Age (months)					0.002
≤12	3032	(24.5)	2608	(25.6)	
13–23	2409	(19.5)	1762	(17.3)	
24–59	6937	(56.0)	5826	(57.1)	
Gender of child					0.242
Male	6045	(48.2)	5077	(49.8)	
Female	6333	(51.2)	5119	(50.2)	
Maternal characteristics					
Maternal age (years)					0.013
15–24	4180	(33.8)	3700	(36.3)	
24–34	5915	(47.8)	4564	(44.8)	
≥ 35	2283	(18.4)	1932	(18.9)	
Educational level					< 0.001
No formal education	1987	(16.1)	1192	(11.7)	
Primary	8310	(67.1)	6708	(65.8)	
Secondary/post-secondary	2081	(16.8)	2296	(22.5)	
Employed					< 0.001
No	2985	(24.1)	2969	(29.1)	
Yes	9394	(75.9)	7227	(70.9)	
Ethnicity					0.289
Chewa	4326	(35.0)	3566	(35.0)	
Tumbuka	1081	(8.7)	938	(9.2)	
Lomwe	2014	(16.3)	1855	(18.2)	
Yao	1634	(13.2)	1498	(14.7)	
Ngoni	1449	(11.7)	1154	(11.3)	
Others	1874	(15.1)	1186	(11.6)	
Marital status					< 0.001
Unmarried	1366	(11.0)	1430	(14.0)	
Married	11,012	(89.0)	8766	(86.0)	
Religion					0.058
Catholic	2655	(21.4)	1900	(18.6)	
Protestant	2581	(20.9)	2279	(22.4)	
Muslim and others	7142	(57.7)	6017	(59.0)	
Health behaviour					< 0.001
Desirable	7889	(64.0)	4447	(36.0)	
Undesirable	4489	(43.9)	5749	(56.1)	
Household factors					
Wealth					0.188
Poorest	2303	(52.1)	2118	(47.9)	
Poorer	2643	(53.6)	2284	(46.4)	
Middle	2786	(27.3)	2076	(42.7)	
Richer	2337	(55.0)	1912	(45.0)	
Richest	2309	(56.1)	1806	(43.9)	
Gender of head of household					< 0.001
Male	10,105	(81.6)	7868	(77.2)	
Female	2273	(18.4)	2328	(22.8)	
Roofing material					< 0.001
Unimproved	8418	(68.0)	5967	(58.5)	
Improved	3960	(32.0)	4229	(41.5)	
Region					0.981
Northern	1430	(11.6)	1167	(11.4)	
Central	5251	(42.4)	4279	(42.0)	
Southern	5697	(46.0)	4750	(46.6)	
Residence					0.830
Urban	1749	(14.1)	1393	(13.7)	
Rural	10629	(85.9)	8803	(86.3)	
Household ITN supply					0.089
Very poor	6007	(56.0)	4718	(44.0)	
Poor	3491	(54.4)	2930	(45.6)	
Good	2880	(53.1)	2548	(46.9)	

*ITN* insecticide-treated net

### Distribution of participants according to ITN usage

The rate for ITN use was 57.8% (95% Confidence interval (CI) 56.1%–59.4%) in 2010 compared to 69.0% (95% CI 67.4%–70.5%) in 2015 (Table 2). Table 2 further reveals that in both survey years, ITN usage was associated with a child being ≤ 12 months old, the mother being aged ≤ 24 years, with secondary or post-secondary education, who was married, with desirable health behaviours, and who came from the richest household, living in a male headed household, in urban residence, and with a good ITN supply (*p *< 0.05). Significant (*p *< 0.05) differences were also observed between ITN users and non-users in terms of ethnicity in both survey waves. However, having employed mother was associated with ITN usage in 2015–2016 only, while belonging to catholic religious group was associated with ITN usage in 2010 but not in 2015–2016.

**Table 2:** Comparison of characteristics by insecticide-treated net (ITN) usage across survey waves

Variable	2010 *n* = 12,378		*p* value	2015–2016 *n* = 10,196		*p* value ^a^
	*n* (%) Yes *n* = 7151	*n* (%) No *n* = 5227		*n* (%) Yes *n* = 7033	*n* (%) No *n* = 3163	
Child factors						
Age (months)			< 0.001			< 0.001
≤ 12	1914 (63.1)	1118 (36.9)		1965 (75.3)	643 (24.7)	
13–23	1463 (60.7)	946 (39.3)		1297 (73.6)	465 (26.4)	
24–59	3774 (54.4)	3163 (45.6)		3771 (64.7)	2055 (35.3)	
Gender of child			0.202			0.917
Male	3446 (57.0)	2599 (43.0)		3506 (69.0)	1571 (31.0)	
Female	3705 (58.5)	2628 (41.5)		3527 (68.9)	1592 (31.1)	
Maternal characteristics						
Maternal age (years)			0.012			0.038
15–24	2431 (58.2)	1749 (41.8)		2617 (70.8)	1083 (29.2)	
24–34	3497 (59.1)	2418 (40.9)		3138 (68.8)	1426 (31.2)	
≥ 35	1223 (53.6)	1060 (46.4)		1278 (66.1)	654 (33.9)	
Educational level			< 0.001			< 0.001
No formal education	1009 (50.8)	878 (49.2)		712 (59.7)	480 (40.3)	
Primary	4702 (56.6)	3608 (43.4)		4552 (67.9)	2156 (32.1)	
Secondary/Post-secondary	1440 (69.2)	641 (30.8)		1769 (77.0)	527 (23.0)	
Employed			0.131			0.004
No	1784 (59.8)	1201 (40.2)		2131 (71.8)	838 (28.2)	
Yes	5367 (57.1)	4027 (42.9)		4902 (67.8)	2325 (32.2)	
Ethnicity			0.019			0.043
Chewa	2529 (58.4)	1797 (41.6)		2380 (66.7)	1186 (33.3)	
Tumbuka	565 (52.2)	516 (47.8)		663 (70.7)	275 (29.3)	
Lomwe	1125 (55.9)	889 (44.1)		1307 (70.5)	547 (29.5)	
Yao	904 (55.3)	730 (44.7)		1017 (67.9)	481 (32.1)	
Ngoni	882 (60.9)	567 (39.1)		855 (74.1)	299 (25.9)	
Others	1146 (61.1)	728 (38.9)		811 (68.3)	375 (31.7)	
Marital status			< 0.001			0.002
Unmarried	670 (49.1)	696 (50.9)		919 (64.3)	511 (35.7)	
Married	6481 (58.9)	4531 (41.1)		6114 (69.7)	2652 (30.3)	
Religion			0.001			0.361
Catholic	1590 (59.9)	1065 (40.1)		1351 (71.1)	549 (28.9)	
Protestant	1587 (61.5)	994 (38.5)		1567 (68.8)	712 (31.2)	
Muslim and others	3974 (55.6)	3168 (44.4)		4115 (68.4)	1902 (31.6)	
Health behaviour			< 0.001			<0.001
Undesirable	4363 (55.3)	3526 (44.7)		2887 (64.9)	1560 (35.1)	
Desirable	2788 (62.1)	1701 (37.9)		4146 (72.1)	1603 (27.9)	
Household factors						
Wealth			< 0.001			< 0.001
Poorest	1159 (50.3)	1144 (49.7)		1372 (64.8)	746 (35.2)	
Poorer	1394 (52.8)	1249 (47.2)		1531 (67.0)	753 (33.0)	
Middle	1633 (58.6)	1153 (41.4)		1415 (68.2)	661 (31.8)	
Richer	1392 (59.6)	945 (40.4)		1330 (69.6)	582 (30.4)	
Richest	1573 (68.1)	736 (31.9)		1385 (76.7)	421 (23.3)	
Gender of head of household			< 0.001			0.003
Male	5997 (59.3)	4108 (40.7)		5507 (70.0)	2361 (30.0)	
Female	1154 (50.8)	1119 (49.2)		1526 (65.6)	802 (34.0)	
Roofing material			< 0.001			0.001
Unimproved	4690 (55.7)	3728 (44.3)		3996 (67.0)	1971 (33.0)	
Improved	2461 (62.1)	1499 (37.9)		3037 (71.8)	1192 (28.2)	
Region			0.127			0.289
Northern	768 (53.7)	662 (46.3)		828 (71.0)	339 (29.0)	
Central	3022 (57.6)	2229 (42.4)		2896 (67.7)	1383 (32.3)	
Southern	3361 (59.0)	2336 (41.0)		2208 (69.7)	1442 (30.3)	
Residence			< 0.001			< 0.001
Urban	1197 (68.4)	552 (31.6)		1111 (79.7)	282 (20.3)	
Rural	5954 (56.0)	4675 (44.0)		5922 (67.3)	2881 (32.7)	
Household ITN supply			< 0.001			< 0.001
Very poor	2849 (47.4)	3158 (52.6)		2696 (57.2)	2022 (42.8)	
Poor	2232 (63.9)	1259 (36.1)		2209 (75.4)	721 (24.6)	
Good	2070 (71.9)	810 (28.1)		2128 (83.5)	420 (16.5)	

^a^All p values were determined by a Chi-square test

### Factors associated with ITN usage

The odds of ITN usage in the 2010 MDHS were lower among children aged ≥ 24 months (aOR = 0.7, 95% CI 0.6–0.8), those whose mother had no formal education or primary education (aOR = 0.7, 95% CI 0.6–0.9 and aOR = 0.8, 95% CI 0.7–0.9, respectively), those with unmarried mothers (aOR = 0.8, 95% CI 0.7–0.9), those living female headed households (aOR = 0.8, 95% CI 0.7–0.9) compared to their respective reference groups (Table 3).

**Table 3:** Multivariate analyses of factors associated with insecticide-treated net (ITN) usage among children under 5 years old

Variable	2010			2015–2016		
	aOR	95% CI	*p* value	aOR	95% CI	*p* value
Child factors						
Age (months)						
≤ 12	1.0			1.0		
13–23	0.9	(0.8–1.1)	0.078	0.9	(0.8–1.1)	0.353
24–59	0.7	(0.6–0.8)	< 0.001	0.6	(0.5–0.7)	< 0.001
Gender of child						
Male	1.0			1.0		
Female	1.1	(0.9–1.2)	0.050	1.0	(0.9–1.1)	0.899
Maternal characteristics						
Maternal age (years)						
15–24	1.0			1.0		
24–34	1.2	(1.0–1.3)	0.042	1.1	(0.9–1.3)	0.349
≥ 35	1.1	(0.9–1.3)	0.214	1.1	(0.9–1.4)	0.177
Educational level						
Secondary/post-secondary	1.0			1.0		
No formal education	0.7	(0.6–0.9)	0.002	0.6	(0.49–0.8)	0.001
Primary	0.8	(0.7–0.9)	0.011	0.8	(0.67–0.9)	0.008
Employed						
No	1.0			1.0		
Yes	0.9	(0.8–1.1)	0.641	0.9	(0.8–1.0)	0.076
Ethnicity						
Lomwe	1.0			1.0		
Chewa	1.3	(1.1–1.7)	0.019	0.9	(0.7–1.2)	0.501
Tumbuka	0.9	(0.7–1.3)	0.685	0.9	(0.7–1.3)	0.622
Yao	1.1	(0.8–1.3)	0.688	0.9	(0.8–1.3)	0.927
Ngoni	1.4	(1.1–1.7)	0.008	1.1	(0.9–1.5)	0.265
Others	1.3	(1.1–1.6)	0.002	0.8	(0.7–1.1)	0.177
Marital status						
Married	1.0			1.0		
Unmarried	0.8	(0.7–0.9)	0.013	0.8	(0.7–1.0)	0.089
Religion						
Catholic	1.0			1.0		
Protestant	0.9	(0.8–1.2)	0.923	0.8	(0.6–0.9)	0.043
Muslim and other	0.9	(0.8–1.1)	0.253	0.9	(0.8–1.1)	0.224
Health behaviour						
Undesirable	1.0			1.00		
Desirable	1.2	(1.1–1.3)	0.001	1.2	(1.1–1.3)	0.016
Household factors						
Wealth						
Poorest	1.00			1.00		
Poorer	0.9	(0.8–1.2)	0.861	1.0	(0.8–1.2)	0.970
Middle	1.2	(1.1–1.6)	0.012	1.0	(0.9–1.3)	0.725
Richer	1.4	(1.1–1.7)	0.004	0.9	(0.8–1.3)	0.944
Richest	1.7	(1.3–2.2)	< 0.001	0.8	(0.6–1.2)	0.275
Gender of head of household						
Male	1.0			1.0		
Female	0.8	(0.7–0.9)	0.002	0.8	(0.7–0.9)	0.012
Roofing material						
Improved	1.0			1.0		
Unimproved	1.4	(1.1–1.7)	0.001	1.0	(0.8–1.2)	0.910
Region						
Northern	1.0			1.0		
Central	1.2	(0.9–1.6)	0.237	0.8	(0.6–1.1)	0.261
Southern	1.5	(1.2–1.9)	0.002	0.9	(0.8–1.3)	0.973
Residence						
Urban	1.0			1.0		
Rural	0.8	(0.6–1.1)	0.060	0.6	(0.5–0.8)	0.001
Household ITN supply						
Very poor	1.0			1.0		
Poor	1.9	(1.6–2.1)	< 0.001	2.3	(1.9–2.6)	< 0.001
Good	2.6	(2.3–2.9)	< 0.001	3.8	(3.2–4.5)	< 0.001

*aOR* adjusted odds ratio, *CI* confidence interval, *ITN* insecticide treated nets

In addition, a higher odds of ITN use was observed in those from Chewa, Ngoni, and other ethnic groups (aOR = 1.3, 95% CI 1.1–1.7; aOR = 1.4, 95% CI 1.1–1.7; and aOR = 1.3, 95% CI 1.1–1.6, respectively), whose mothers had desirable health behaviours (aOR = 1.17, 95% CI 1.04–1.31), middle, richer, and richest households (aOR = 1.2, 95% CI 1.1–1.6, aOR = 1.4, 95% CI 1.1–1.7 and aOR = 1.7, 95% CI 1.3–2.2, respectively), from households with unimproved roofing material (aOR = 1.4, 95% CI 1.1–1.7), from the southern region (aOR = 1.48, 95% CI 1.16–1.89), and from households with poor and good ITN supply (aOR = 1.9, 95% CI 1.6–2.1 and aOR = 2.6, 95% CI 2.3–2.9, respectively) compared to their respective reference groups (Table 3).

In contrast, in 2015–2016, ethnicity, marital status, wealth, roofing material, region, and health promotion did not exhibit significant associations with ITN usage. In 2015–2016, children whose mothers had desirable health behaviours were more likely to sleep under an ITN (aOR = 1.2, 95% CI 1.1–1.3) compared to those whose mothers did not have desirable health behaviours. Additionally, in 2015–2016, belonging to the Protestant religious group and residing in rural areas were associated with reduced likelihood of ITN usage (aOR = 0.8, 95% CI 0.6–0.9) and (aOR = 0.6, 95% CI 0.5–0.8) compared to Catholics and urban dwellers, respectively (Table 3).

## Discussion

This is the first nationally representative study to compare ITN usage in children under 5 years old living in households that owned at least one ITN between 2010 and 2015 following the nationwide ITN distribution campaign in 2012. Despite similar ITN access rates between the two surveys (38% for 2010 and 39% for 2015) [12], the rate of ITN usage in children under 5 years old increased from 57.8% in 2010 to 69.0% in 2015. Moreover, the study revealed that many child, maternal and household factors were significantly associated with ITN usage across the two surveys. Specifically, children whose mothers had desirable health behaviours, from households with a good ITN supply, and living in the southern region had increased odds of using ITNs.

The increase in ITN usage observed in this study could probably be attributed to two reasons; First, the nationwide ITN distribution campaign in 2012, and a partial distribution campaign in early 2015, which were accompanied by awareness messages on the importance of using malaria interventions such as ITNs. In 2015, a higher proportion (32%) of the interviewed households reported that they sourced their ITNs from mass campaigns than any other sources [12]. Second, the differences in survey periods (i.e. the 2015 survey was largely conducted during malaria transmission peak period (October–February) while most part of the 2010 survey was conducted outside the transmission peak period (June–November). Despite the observed increase in ITN usage, the Malawian ITN usage rate remains relatively lower than that in other countries in SSA such as Rwanda (75%) [18]. The observed difference in ITN usage rates may be as a result of differences in ITN access rates at the time of the study with Rwanda reporting a relatively high ITN access of 64.2% compared to the 39% for Malawi [19]. Additionally, the rates of households owning at least one ITN in Malawi have been fluctuating from 57% in 2010, 70% in 2014, and 57% in 2015 with high rates of ownership observed in the years soon after mass ITN campaigns [12]. The results suggest the need for continued efforts in increasing household ITN ownership and access, through strengthened mass ITN distribution campaigns and routine distribution systems to vulnerable groups.

Children whose mothers were married, had desirable health behaviours, those from middle, rich and richest households, and from households with unimproved roofing were more likely to sleep in an ITN in 2010. Unmarried women may lack support from their spouses and may be disadvantaged in having access to resources [20]. Previous research assessing the association between socio-economic status and net usage revealed conflicting results [5, 21–23]. Mothers from rich and middle-wealth households may have better access to information and resources that may help them reinforce the decision to have their children sleep under an ITN [24]. Consistent with a previous study in Ethiopia, having improved roofing in a household may foster a false sense of protection from mosquitoes, and hence families may be more likely to forego nets [25]. Women already practicing good health behaviours might be more receptive to other health-promotion practices, such as the use of ITNs [26]. Ethnicity and geographical regions were highly correlated with cultural beliefs and social norms which may influence health behaviours [27]. A higher malaria prevalence was reported in the southern region than the northern part of Malawi [28]; hence, people in the south may feel vulnerable to malaria, thereby making greater efforts to use intervention strategies already in place [28].

The diminishing influence of these aforementioned factors could be related to the mass ITN distribution campaign that was conducted in 2012, which also included a comprehensive awareness campaign. It further appears that the campaign achieved some moderate success in alleviating the influence of socio-economic factors on net usage, as well as in minimizing cultural and regional differences in net usage. This underscores the importance of mass campaigns in reducing disparities in ITN usage behaviour among various segments of the population in Malawi. Future mass campaigns should, therefore, not only emphasize ITN distribution, but also strengthen behavioural change communication messages related to ITN usage.

Additionally, data indicated that older children (≥ 24 months) were less likely to sleep in an ITN compared to those aged ≤ 12 months. It was shown that as children grow older, their access to ITN is lost to younger siblings [29]. Moreover, younger children, especially those that are breastfeeding, are more likely to share a bed with their parents, thereby increasing their chances of sleeping under an ITN [21]. Hence, there is a need to holistically enhance ITN usage among all age groups of children to achieve the malaria elimination goal in Malawi. Mother’s education remained a significant factor, consistent with previous research [30]. A mother’s level of education may portray the level of autonomy the mother has on important issues such as health decisions [31]. Autonomous women were associated with improved health behaviours [32, 33] which may subsequently affect the behaviour of their children [34].

Children from households headed by a female were associated with reduced odds of ITN usage. Households headed by a female may have limited access to resources [35], such as radios, newspapers and television to expose them to important health messages compared to households headed by a male, which may ultimately play a role in their health behaviours, including ITN usage for their younger ones. In addition, a male’s position on ITNs might influence household ITN usage as reported in Liberia [36]. Similarly, rural residents might have poor access to health-related information and other resources [3, 25, 37], hence this explains the finding that children from rural areas being less likely to use ITN compared to urban residents. Moreover, households in rural areas might also face problems of limited public health resources and administrative support within the community.

ITN usage is largely dependent on access [13]. This study demonstrated increased odds of ITN usage among children from households with good ITN supply compared to those with very poor ITN supply. Compared to smaller households, larger households face challenges in achieving desirable intra-household ITN supply [38]. This finding underscores the need for malaria programmes to take into consideration family sizes during ITN distribution which may help increase intra-household ITN supply and, ultimately, help improve usage.

In 2015, those belonging to the Protestant religious group had reduced odds of using ITN compared to belonging to the Catholic religious group. This is consistent with a previous study in Ghana where Catholics were more likely to use ITNs compared to those who practiced traditionalism [39]. Efforts to enhance ITN utilization should consider partnership with community religious institutions as an essential approach to scaling up ITN utilization by children.

There are several limitations to this study that should be noted. First, because of the limited number of variables in the MDHS, an array of other important factors related to ITN usage, particularly user-side factors, such as satisfaction with the nets or difficulties associated with using the nets, were not examined. However, this study captured many important child, maternal and household characteristics that could prove vital for effective ITN programmes. Second, the cross-sectional design limited the study from drawing causal inferences between explanatory factors and ITN usage. Third, the study used wealth quintiles which were calculated using PCA model and not multiple component analysis which has been shown to provide more accurate wealth index because of its ability to accommodate both continuous and dichotomous variables [40]. The inability of PCA analysis to accommodate continuous variables makes it difficult to determine wealth quintile cut-off points where most households have the same assets which may result in allocating the same or very similar wealth scores [40]. Finally, the question on ITN usage by children was asked from the mother which could be subject to social desirability bias as mothers may want to prove that they are taking good care of their children by making them sleep under ITNs. Despite these limitations, the study provides evidence of the importance of considering child, maternal and household characteristics, in both research and actual practice, to promote the use of ITNs.

## Conclusions

This study examined associations between ITN usage and a comprehensive list of child, maternal and household characteristics. Results demonstrated a considerable increase in the rates of ITN usage among children under 5 years old in Malawi following a nationwide ITN distribution campaign in 2012. However, more should be done to increase ITN usage, and public health practitioners should consider factors identified in this study when distributing and promoting ITN usage in Malawi.

## Abbreviations

ITN: insecticide-treated nets
aOR: adjusted odds ratio
CI: confidence interval
EA: enumeration area
PCA: principle component analysis
SSA: sub-Saharan Africa
DHS: Demographic Health Survey
MDHSs: Malawi Demographic Health Surveys
ICF: International Classification of Functioning Disability and Health
MPHC: Malawi Population and Housing Census
